# A comparative study of learners' conceptions of and approaches to learning English between high school students in urban and rural areas of China

**DOI:** 10.3389/fpsyg.2024.1324366

**Published:** 2024-08-23

**Authors:** Huilin Fu, Hanyong Liu

**Affiliations:** ^1^School of Humanities, Beijing University of Posts and Telecommunications, Beijing, China; ^2^Shenzhen Songgang Middle School, Shenzhen, China

**Keywords:** conceptions of learning English, approaches to learning, English learning, high school students, urban and rural areas

## 1 Introduction

The differentiating developmental status of teaching and learning in rural areas is noteworthy and of great significance for the education equity. Taking People's Republic of China as an example, it has the largest population in the world and a large percentage of people living in rural areas. According to the 7th national census data released by the National Bureau of Statistics of China in 2021, the total population of China was 141.178 million by 2020. The rural population was 509.79 million, accounting for 36.11% of the whole population (National Bureau of Statistics, 2021). The socio-economic development in different parts of China is unbalanced, and disparity still exists between developed provincial capital cities and remote western inland areas (Song et al., 2020). It may further enlarge the gap between urban and rural educational development, which had washback effects on restricting the talent development in local areas. The realization of social equity and justice affected the construction and development of a harmonious society in China (Zhao, 2011). Due to the different economic status in urban and rural areas in China, students in urban or rural schools may have different accesses to digital resources or facilities. The uneven distribution of educational resources between urban and rural schools may lead to further differences in learning outcomes.

Lots of attention has been paid to the role of learners' conceptions of and approaches to learning were significant factors influencing learners' learning outcomes. Conceptions of learning guide students' primary beliefs about the experiences of learning as well as their interpretations of learning itself and have been found to be strongly related to learning outcomes (Chin and Brown, 2000; Tsai, 2004). Approaches to learning are also seen as powerful means of modeling student learning and the quality of learning outcomes (Prosser and Millar, 1989; Trigwell and Prosser, 1991; Umapathy et al., 2020). Furthermore, it has been proved that students' conceptions of learning are closely related to approaches to learning (Chin and Brown, 2000; Liang et al., 2015; Yang et al., 2019). For example, previous research has found that students with lower-level conceptions of learning were more likely to use surface approaches to learning, while students possessing higher-level conceptions of learning tended to adopt deep approaches to learning (Chiou et al., 2013; Liang et al., 2015). In addition, findings of previous literature suggested that students' conceptions of and approaches to learning were socially and culturally dependent (Tsai, 2004; Kember and Watkins, 2010).

Students' conceptions of and approaches to learning have been explored in different disciplines, such as mathematics (Cai et al., 2018), science (Lee et al., 2008; Lin and Tsai, 2013), computer science (Liang et al., 2015; Umapathy et al., 2020), engineering (Ellis et al., 2008), chemistry (Li et al., 2013), programming (Chou et al., 2021), biology (Google et al., 2023) and physics (Cai et al., 2021). However, previous studies concerning those two concepts rarely take urban and rural differences into account and very few of them focus on language learning. And there is only a limited number of studies investigating the relationships between language learners' conceptions of learning and their approaches to language learning. Thus, it is of great significance to focus on urban and rural learners and further explore their conceptions of and approaches to learning English.

High school education is an important part of the national education system and plays a key role in talent training of China. As an important link connecting higher education and compulsory education, senior high school English education plays an important role in the whole English education system (Manxia, 2018). The population of senior high school students in China has reached approximately 40 million (Ministry of Education of People's Republic of China, 2022), and for most senior high schools, English is a compulsory course. Given this large population of high school students and the importance of learning English well in high school, investigating high school English learners' conceptions of and approaches to learning English is of great significance. In the current education system of all levels in China, high school education still faces many difficulties and challenges, especially in rural areas (Yu et al., 2023) or poor areas of central and western China (Xu, 2020). Thus, it is meaningful to further understand high school students' conceptions of learning and approaches to learning English in urban and rural areas in China. This research was set in two high schools in Guizhou and Beijing, focusing on the similarities and differences of learners' conceptions of and approaches to learning English among high school students in urban and rural areas, as well as the associations between these two constructs. It is believed that the research findings may have some implications for English education in less-developed rural areas, narrowing educational disparities and contributing to the broader field of language education with joint efforts.

## 2 Literature review

### 2.1 Conceptions of learning

Conceptions of learning were drawn from individuals' learning experiences, referring to students' views on, understanding of, or beliefs about their learning objects and process and their preferred ways of undertaking the learning process (Benson and Lor, 1999; Tsai, 2004; Liang et al., 2010; Zheng et al., 2016, 2018; Tao et al., 2020). Students' conceptions of learning have been found to be strongly related to the learning process (Lee et al., 2008; Sadi and Lee, 2015), and therefore to learning outcomes (Chin and Brown, 2000; Tsai, 2004). The conceptions of learning were firstly proposed by Saljö (1979). He distinguished five qualitatively different categories of conceptions of learning, including “memorizing”, “increase of knowledge”, “acquisition of facts, procedures that can be retained and utilized in practice”, “abstraction of meaning”, and “an interpretative process aimed at the understanding of reality”. Then, Marton et al. (1993) developed a framework of six conceptions of learning: “memorizing and reproducing”, “increase of knowledge”, “applying information”, “understanding”, “seeing something in a different way” and “changing as a person”, in which the first three conceptions were described as constituting a reproductive conception of learning, whereas the latter three were considered to represent a constructivist view (Marton et al., 1993). Following Saljö and Marton, many researchers have investigated the conceptions of learning held by students in a variety of educational contexts, varying from elementary to higher education levels, and subject domains including engineering, mathematics, science, physics, and so on (e.g., Eklund-Myrskog, 1998; Marshall et al., 1999; Morris, 2001; Tsai, 2004, 2009; Duarte, 2007; Lin and Tsai, 2008; Zheng et al., 2016, 2018).

Conceptions of learning also follow a hierarchical order (Marton, 1994; Tsai, 2004; Lin et al., 2012; Soltani and Askarizadeh, 2021). A constructivist-reproductive distinction has been identified that students with lower-level (reproductive) conceptions perceived learning as involving accumulation and memorization of often isolated factual knowledge, largely for assessment purposes. On the contrary, learners with higher-level (constructivist) conceptions have an internalized focus and see learning as a process of understanding, integrating and deriving personal meaning and then achieve better academic outcomes (Marton et al., 1993; Richardson, 2011; Liang et al., 2015; Umapathy et al., 2020). Conceptions of learning were classified in increasingly complex categories, starting from memorizing and knowledge acquisition, then applying knowledge, understanding, or making sense of knowledge, and finally seeing knowledge from new perspectives or changing as a person or creating new knowledge (Lonka et al., 2020).

Tsai (2004) proposed a framework for conceptions of learning computer science with seven categories named “Memorizing”, “Preparing for Tests”, “Calculating and Practicing Tutorial Problems”, “The Increase of Knowledge”, “Applying”, “Understanding”, and “Seeing in a New Way”. The author identified the first three (“memorizing”, “preparing for tests”, and “calculating and practicing tutorial problems”) as lower-level (reproductive) conceptions of learning, and the last four (“the increase of knowledge”, “applying”, “understanding”, and “seeing in a new way”) as higher-level (constructivist) conceptions of learning. In the field of second language learning (SLA), conceptions of learning refer to language learners' beliefs about what a foreign language is, and what the language learning process consists of Benson and Lor (1999). Zheng et al. (2016) developed a COLE questionnaire to investigate college students' conceptions of learning English and identified eight factors of learners' conceptions of learning English, named “Memorizing”, “Testing”, “Drill and Practice”, “Grammar, Vocabulary, and Pronunciation”, “Increasing One's Knowledge”, “Application and Communication”, “Understanding” and “Seeing in a New Way”. And the validity of this classification was also proved in later research (Luan and Zheng, 2017; Tao et al., 2020).

### 2.2 Approaches to learning

Approaches to learning refer to students' ways of experiencing and managing learning situations and are defined as a fusion of motivation on learning and the use of appropriate strategies by students (Zhang and Stenberg, 2000; Biggs, 2001) or “the ways in which students go about their academic tasks, thereby affecting the nature of the learning outcomes” (Biggs and Telfer, 1987). Approaches to learning were seen as powerful means of modeling student learning and the quality of learning outcomes (Prosser and Millar, 1989; Trigwell and Prosser, 1991). Basically, two approaches to learning have been identified: the “surface approach” and the “deep approach” (Marton and Saljö, 2005). And following studies verified the feasibility of this categorization (e.g., Chin and Brown, 2000; Liang et al., 2010; Lin et al., 2012; Vanthournout et al., 2014; Asikainen and Gijbels, 2017). In addition, Biggs (1987) identified achievement approach as a third learning approach, and Roshanaei (2023) related surface, achieving and deep approaches with preferences for instructions, the while in this research the deep and surface approaches were mainly focused on.

The general framework and defining features of the deep and surface approaches were described by Marton (1983) and Biggs (1987). The deep approaches were associated with intrinsic motivation and interest in the content of the task, focusing on understanding the meaning of the learning material and engaging in meaningful learning (Biggs and Tang, 2007; Asikainen and Gijbels, 2017), while the surface approaches were based on extrinsic or instrumental motivation, perceiving the task as a demand to be met and tending to just pass assessment, and to fulfill the minimum requirements through rote learning (Chin and Brown, 2000; Biggs and Tang, 2007; Vanthournout et al., 2014). Accordingly, the surface learning approaches were then characterized as surface motivation (extrinsic motivation such as fear of failure) and surface strategies (solely memorizing the parts needed to pass the examinations). In contrast, the deep learning approach consisted of deep motivation (intrinsic motivation such as inner interest) and deep strategies (understanding the main ideas thoroughly or using comprehensive ways to learn) (Chiou and Liang, 2012).

Relationships between academic success and approaches to learning have been well discussed. Previous studies have proved that learners who adopted surface approaches tended to have lower academic outcomes and were less successful in school, whereas those with a deep approach to learning are more successful in school (Gynnild and Myrhaug, 2012; Arquero et al., 2015; Rozgonjuk et al., 2018). In other words, students with deep learning approaches were likely to make greater progress and have a better understanding of the subject compared to those with surface approach to learning (Micari and Light, 2009; Yang and Tsai, 2010; Purwanto and Pratiwi, 2017). In the field of second language learning, previous research has provided the evidence of a relationship between a surface approach to learning English and a lower level of English ability among EFL learners. For example, Gow et al. (1991) found that students who were weaker in English tended to use a surface approach to learning. Moreover, it was found that deep approaches and surface approaches to learning English were also significantly correlated (Magno, 2009).

### 2.3 Relationship between conceptions of and approaches to learning

It has been proved that students' conceptions of learning were closely related to approaches to learning (Liang et al., 2010, 2015; Richardson, 2011; Chiou et al., 2013; Shen et al., 2016; Huang et al., 2018; Yang et al., 2019; Mulyani et al., 2020). A large number of studies concerning approaches to learning were discussed with conceptions of learning (e.g., Serife, 2008; Chiou et al., 2013; Liang et al., 2015; Monroy and Gonzalez-Geraldo, 2018). And it was believed that students with lower-level conceptions of learning were more likely to use surface approaches to learning, while students possessing higher-level conceptions of learning tended to adopt deep approaches to learning (Lee et al., 2008; Chiou et al., 2012, 2013; Liang et al., 2015; Yang et al., 2019). If students hold lower-level conceptions of learning, such as perceive learning as rote memorization of facts, it is likely that they will tend to adopt a surface approach to learning, while those who possess higher-level conceptions of learning and conceive learning to be about meaning and understanding, are likely to adopt a deep approach which centers on understanding (Liang et al., 2015; Tsai et al., 2016; Umapathy et al., 2020). Chiou et al. (2013) found that students' higher-level learning conceptions such as “seeing problems in a new way” were more likely to positively correlate with their deep approaches to learning physics, whereas their lower-level conceptions of learning such as “testing” were more likely to correlate with their surface approaches and negatively predict students' deep approaches to learning physics. Umapathy et al. (2020) also revealed that students with higher-level conceptions of learning (such as seeing in a new way) tended to adopt deep approaches in learning computer science, while learner holding lower-level conceptions of learning (such as Memorizing) were more associated with surface approaches to learning computer science. In a word, conceptions of learning influenced the approaches to learning, and approaches to learning consequently influenced learning outcomes (Yang and Tsai, 2010). There are rather complex relationships between conceptions of and approaches to learning, which requires researchers and educators to investigate and explore the intricate relationships with empirical studies in different contexts and disciplines.

### 2.4 The role of social-economic-culture background in students' learning

The growing socio-economic inequality between different regions has become a major challenge in the 21st century with the global urbanization (Winthrop et al., 2018). It has been generally proved that language learning is significantly and inseparably associated with the social background factors. For examples, socioeconomic background has an important influence on motivational beliefs (King and McInerney, 2016; Ma et al., 2021), level of anxiety during learning (Rafael and Ana, 2016; Yu et al., 2023), and learner engagement (Fredricks et al., 2016; Tian and Yuan, 2019). For instance, Rafael and Ana (2016) figured out that content and language integrated learning involves with aspects which are more susceptible to the influence of the social environment. And this social environment, as the authors referred, covered both urban/rural divide and the socio-economic status (SES) of the parents (families) (Rafael and Ana, 2016). It is noticeable that findings of previous literature suggested that students' conceptions of and approaches to learning are socially and culturally dependent (Tsai, 2004; Kember and Watkins, 2010). For example, Tsai (2004) figured out that Taiwanese students tended to hold a lower-conception of “testing” due to the traditional Chinese exam-oriented culture background in Taiwan. Thus, this research aimed to investigate learners' conceptions of and approaches to learning English in urban and rural settings, and to discuss about the similarities and differences from the perspective of socio-economic-cultural perspective. In addition, the use of surface or deep approaches to learning English was also influenced by the socio-economic backgrounds of the learners (Aharony, 2006; Yang et al., 2019). For example, previous research has proved a clear preference of participants from all socio-economic backgrounds toward the surface learning strategy, and students from higher socio-economic background used both learning strategies more frequently than lower socio-economic students (Aharony, 2006).

### 2.5 Research purposes and research questions

Although a substantial body of research has focused on learners' conceptions of and approaches to learning, studies linking leaners' conceptions of learning with their approaches to learning in the field of language learning were seldom found in existing literature. This study aims to investigate the similarities and differences of learners' conceptions of and approaches to learning English between high school students in urban and rural areas. To fulfill the research purposes, two questionnaires for investigating high school students' conceptions of learning English (COLE) and approaches to learning English (ATLE) were developed to be administered to the high school students investigated in urban and rural areas of China. This research is intended to answer these four questions:

(1) What are the conceptions of learning English among students from two high schools in urban and rural areas of China?
(2) What are their approaches to English language learning?
(3) How do their conceptions of learning English associate with their approaches to learning English?
(4) How do the two research constructs and their associations differ among the two groups of students?

## 3 Methodology

### 3.1 Research context

This research was located at two senior high schools in Beijing and Guizhou, being two typical samples of urban and rural areas in China. These two schools are both outstanding schools in the locality with brilliant teachers, complete teaching facilities and great learning atmosphere. The high school in Beijing is in the central area with abundant educational and social resources. It has complete infrastructure and outstanding teaching facilities, in which each classroom is equipped with perfect multimedia equipment. The high school in Guizhou lies in a poverty-impacted county, being a less-developed region that received the national poverty alleviation subsidies annually by 2020. Over 70% of students in this school being ethnic minorities, the students in this school have relatively limited access to educational resources. Compared with the students in Beijing, students in the rural area have much less access to teaching and learning resources.

### 3.2 Participants

Participants from Beijing and Guizhou were invited to complete the two questionnaires in one setting anonymously and respectively. After eliminating some inadequate data, 616 participants' responses were remained and analyzed. Guizhou participants were 345 Grade Three students, with 237 of them being male students and 108 being female. The age of participants in Guizhou ranged from 17 to 23 years old, with an average of 19.28 years old. 73.62% of them were ethnic minorities including Buyi, Miao, Tujia, Yi, Dong, etc. and the rest were Han nationality. The Beijing participants were 271 Grade Three students, including 140 male and 131 female students. 90.41% of them were Han nationality students. The average age of the Beijing participants was 18.29 years old, ranging from 17 to 21 years old. Before taking part in the study, all the participants had received at least 6 years of formal English education.

### 3.3 Instruments

This research employed two questionnaires to explore the high school students' conceptions of English language learning and their approaches to English language learning. The two questionnaires were adapted from two valid and reliable instruments. The first questionnaire was developed to investigate the students' conceptions of learning English (COLE) survey. It was adapted from the previous questionnaire for assessing college students' conceptions of learning English (Zheng et al., 2016). The second questionnaire was developed to explore leaners' approaches to learning English (ATLE) survey. It was adapted from the previous survey for investigating students' approaches to learning computer science (Chiou and Liang, 2012). All the questionnaire items were measured with a five-point Likert scale, from 1 “do not agree at all” to 5 “strongly agree”. Since English is a foreign language for the participants, all the items in the questionnaires were translated into Chinese and revised by the teachers of the two schools in Beijing and Guizhou. All subitems of the two questionnaires were modified by changing the statements to target high school English language learners more specifically.

### 3.4 Data collection and data analysis

As is mentioned above, the data were collected through two questionnaires for investigating high school students' conceptions of learning English (COLE) and approaches to learning English (ATLE). The above questionnaires were administered among the participants with the permission of the school managers. And the participants in this research volunteered to respond to the two questionnaires in one setting, and all of them completed the two questionnaires anonymously. The procedures of data analysis in this research involved the following phases. First, exploratory factor analysis (EFA) was performed to examine the factor structure and the validity of the factors in these two modified questionnaires using SPSS 25.0. And reliability analysis was also conducted. Second, the descriptive statistics were analyzed to compare learners' mean values and variance of conceptions of and approaches to learning English in urban and rural areas. Third, the correlation between the finalized COLE and ATLE factors was analyzed using Pearson correlation analysis. Moreover, the results were achieved based on *T*-test analysis to check whether there is a significant difference between the mean values of high school learners' conceptions and approaches to learning English in urban and rural areas. Last but not least, stepwise regression was conducted to establish a regression model to describe the specific relationship between learners' conceptions of and approaches to learning English.

## 4 Results

### 4.1 The validity and reliability analysis of COLE and ATLE survey

#### 4.1.1 The validity and reliability analysis of COLE survey

Exploratory factor analysis (EFA) and reliability analysis were conducted to examine the factorial structure and the validity and reliability of the factors in these two modified questionnaires. The statistics were analyzed through SPSS 25.0. The results of the two questionnaires have shown high validity and reliability. Table 1 demonstrates the rotated factor loadings and Cronbach's alpha values for each dimension of the COLE survey, as well as the details of the specific items. The EFA results showed that there were eight factors generated with a total of 38 items. The reliability coefficients for these factors were 0.95 (Meeting the Requirements, MR), 0.87 (Memorizing, Me), 0.89 (Testing, Te), 0.85 (Drills and Practice, DP), 0.85 (Increasing Knowledge, IK), 0.93 (Applying, Ap), 0.91 (Understanding, Und), and 0.96 (Seeing in a New Way, Se). The factor loading of each item was around 0.57–0.86. The overall alpha was 0.84, and the total variance explained was 75.72%. Accordingly, these factors were considered sufficiently reliable to assess the students' conceptions of learning English in urban and rural contexts.

**Table 1:** Validity and reliability data of the COLE survey (*N* = 616).

**Dimensions, items, and descriptive statistics**	**Factor loadings**
**Factor 1: meeting the requirements (MR), mean** = **3.02, S.D**. = **1.04, Cronbach alpha** = **0.95**	
MR-1 I learn English mainly for school requirements.	0.83
MR-2 I learn English mainly for conforming to school curriculum.	0.86
MR-3 I learn English mainly for finishing the school tasks.	0.85
MR-4 I learn English mainly for following the teachers' instructions.	0.80
**Factor 2: memorizing (Me), mean** = **3.10, S.D**. = **0.80, Cronbach alpha** = **0.87**	
Me-1 Learning English is to memorize contents in the textbooks.	0.69
Me-2 Learning English is to memorize what the teacher said in class.	0.72
Me-3 Learning English is to memorize grammars and sentence structures.	0.69
Me-4 Learning English is like learning other liberal arts courses. What is the most important is to memorize the contents of the textbook.	0.73
Me-5 Learning English is to memorize rules of pronunciation.	0.66
**Factor 3: testing (Te), mean** = **2.75, S.D**. = **0.95, Cronbach alpha** = **0.89**	
Te-1 I would not learn English if there were no exams.	0.83
Te-2 Learning English didn't help me much beyond exams, so I could get along fine without English.	0.82
Te-3 I learn English mainly for passing the exams.	0.75
Te-4 I learn English mostly due to exams.	0.61
**Factor 4: drills and practice (DP), mean** = **3.67, S.D**. = **0.68, Cronbach alpha** = **0.85**	
DP-1 Learning English is to practice, including listening, speaking, reading, writing, and translating.	0.78
DP-2 Learning English is to constantly practice how to solve problems.	0.69
DP-3 After I practice a lot, I think I will do better in English classes.	0.70
DP-4 To learn English well, I have to practice pronunciation constantly.	0.57
DP-5 Learning English has a lot to do with repeated practice.	0.71
**Factor 5: increasing knowledge (IK), mean** = **3.72, S.D**. = **0.74, Cronbach alpha** = **0.85**	
IK-1 When teachers are teaching me new words and sentences, I am learning English.	0.74
IK-2 Learning English is to correctly pronounce new words and sentences.	0.58
IK-3 When my English knowledge increases, I think this is English learning.	0.72
**Factor 6: applying (Ap), mean** = **3.59, S.D**. = **0.70, Cronbach alpha** = **0.93**	
Ap-1 Learning English is mainly to understand some foreign materials, like English instructions, to improve the quality of life.	0.62
Ap-2 Learning English is mainly to better communicate with foreigners.	0.77
Ap-3 Learning English is mainly to go abroad for sightseeing.	0.81
Ap-4 Learning English is mainly to go abroad for further study.	0.76
Ap-5 Learning English is mainly to make friends with foreigners.	0.80
Ap-6 Learning English is mainly to appreciate foreign films and television programs.	0.75
Ap-7 Learning English is mainly to make life more convenient.	0.60
Ap-8 Learning English is mainly for future work needs.	0.58
**Factor 7: Understanding (Und), mean** = **3.65, S.D**. = **0.73, Cronbach alpha** = **0.91**	
Und-1 I learn English mainly to understand the rules of grammar and pronunciation.	0.72
Und-2 I learn English mainly to understand the relationships between grammatical concepts.	0.72
Und-3 I learn English mainly to understand the differences between different languages.	0.59
**Factor 8: seeing in a new way (Se), mean** = **3.91, S.D**. = **0.70, Cronbach alpha** = **0.96**	
Se-1 Learning English enables me to understand more cultures and various social phenomena.	0.81
Se-2 I learn English mainly to broaden my horizon.	0.82
Se-3 I learn English to understand social phenomena or things in the world in a new perspective.	0.85
Se-4 Learning English can change my way to understand social phenomena or things in the world.	0.84
Se-5 Learning English is a way for me to understand multiculturalism.	0.81
Se-6 Learning English gives a different outlook on life.	0.79

Overall alpha = 0.84, total variance explained = 75.72%.

#### 4.1.2 The validity and reliability analysis of ATLE survey

Through exploratory factor analysis, a total of 44 items were identified and were further grouped into six factors. Table 2 shows the mean and standard deviation for each item of the ATLE survey, as well as the details of these items. The six factors were named “Aim for Qualification (AQ)” (α = 0.89), “Fear of Failure (FF)” (α = 0.91), “Intrinsic Interest and Commitment to Work (II-CW)” (α = 0.95), “Minimizing Scope of Study (MSS)”, (α = 0.91), “Memorization (Mem)” (α = 0.90), and “Relating Ideas and Understanding (RI-Und)” (α = 0.96). “Aim for Qualification (AQ)” and “Fear of Failure (FF)” constituted “Surface Motive (SM)”; “Intrinsic Interest and Commitment to Work (II-CW)” was “Deep Motive (DM)”; “Minimizing Scope of Study (MSS)” and “Memorization (Mem)” constituted “Surface Strategy (SS)”, while “Relating Ideas and Understanding (RI-Und)” was “Deep Strategy (DS)”. The factor loading of each item was around 0.51–0.86, while the total alpha was 0.87, whose total variance explained was 75.63%, indicating the satisfactory internal consistency of assessing Beijing and Guizhou learners' approaches to learning English.

**Table 2:** Validity and reliability data of the ATLE survey (*N* = 616).

**Dimensions, items and descriptive statistics**	**Factor loadings**
**Surface motive (SM)**	
**Factor 1: aim for qualification (AQ), mean** **=** **3.72, S.D**. **=** **0.67, Cronbach alpha** **=** **0.89**	
AQ-1 Learning English can help me find an ideal job in the future, whether I like it or not.	0.73
AQ-2 I want to get good grades in English learning so that I can find a good job in the future.	0.73
AQ-3 I want to do well in English learning so that my family and teachers can be happy.	0.65
AQ-4 I will try to learn English well in order to enter university.	0.74
AQ-5 Even if I don't like English classes, I will still work hard to get good grades.	0.73
**Factor 2: fear of failure (FF), mean** **=** **3.47, S.D**. **=** **0.78, Cronbach alpha** **=** **0.91**	
FF-1 When I don't do well in the English exam, I will worry about my performance in the next exam.	0.71
FF-2 Although I work hard to prepare for the English exam, I still worry about not doing well in the exam.	0.80
FF-3 I will worry that my performance in English class does not meet the teacher's expectations.	0.73
FF-4 I'm still afraid of learning English although I try to relax.	0.75
FF-5 Whenever I take an English test, I always worry that I don't do well.	0.83
FF-6 Whenever I take an English test, I feel nervous and afraid.	0.78
**Deep motive (DM)**	
**Factor 3: intrinsic interest and commitment to work (II-CW), mean** **=** **3.48, S.D**. **=** **0.70, Cronbach alpha** **=** **0.95**	
II-CW-1 When learning English, I often feel very happy and satisfied.	0.78
II-CW-2 When learning English, I am always interested in the content.	0.78
II-CW-3 Learning English is very interesting, so I will study English very hard.	0.79
II-CW-4 I am always looking forward to having English classes.	0.77
II-CW-5 I like what the teacher teaches in English class now.	0.69
II-CW-6 The learning content in English textbooks is very attractive.	0.73
II-CW-7 I will spend my spare time studying the contents related to English courses on my own.	0.71
II-CW-8 Before having an English class, I always have a lot of questions in my mind.	0.73
II-CW-9 Even when I take other courses, I still have English-related content in my mind.	0.65
II-CW-10 I like to work hard to study English and sum up, which will make me feel very successful.	0.72
II-CW-11 I will seriously complete the exercises in the textbook even if it is not required.	0.68
**Surface strategy (SS)**	
**Factor 4: minimizing scope of study (MSS), mean** **=** **3.11, S.D**. **=** **0.80, Cronbach alpha** **=** **0.91**	
MSS-1 When learning English, I won't pay attention to what is unrelated to the exams.	0.77
MSS-2 I will reduce the time of English learning as long as I can cope with the exam.	0.83
MSS-3 There are too many contents to learn in high school. I will treat them differently and will not spend too much time and energy on English learning.	0.84
MSS-4 English learning involves many contents, so I won't be too familiar with of each unit.	0.87
MSS-5 I hope that the teacher can draw out the key points of the exam so that I can prepare for the exam.	0.64
MSS-6 If the scope of the English test is too wide, I will choose to give up or just do the simple part.	0.73
**Factor 5: memorization (Mem), mean** **=** **2.87, S.D**. **=** **0.909, Cronbach alpha** **=** **0.90**	
Mem-1 The best way to score high in English exams is to memorize the answers of exercises.	0.76
Mem-2 As long as I memorize the important contents in the English textbook, I can get high marks in most exams even if I don't understand them.	0.74
Mem-3 I will pay special attention to memorizing, especially the possible part of English exams.	0.58
Mem-4 To score high in the English exams, memorizing is more important than understanding.	0.75
**Deep strategy (DS)**	
**Factor 6: relating ideas and understanding (RI-Und), mean** **=** **3.63, S.D**. **=** **0.68, Cronbach alpha** **=** **0.96**	
RI-Und-1 When learning English, I will try to relate what I have learned with other units or subjects.	0.73
RI-Und-2 When learning English, I like to relate some scattered learning contents together.	0.75
RI-Und-3 When learning English, I will try to find out the connections among what I have learned.	0.79
RI-Und-4 When learning new contents in English textbooks, I will relate them with what I have learned.	0.78
RI-Und-5 I will relate my previous knowledge to help me finish my English homework.	0.78
RI-Und-6 When preparing for exams, I will relate what the teacher has said with my review materials.	0.75
RI-Und-7 When learning new English words, I will consider the application situations of these words.	0.77
RI-Und-8 When learning English, I will try to understand the teacher's lecture as much as possible.	0.78
RI-Und-9 When I read English textbooks, I will try to understand the meaning behind these contents.	0.73

Overall alpha = 0.96, total variance explained = 75.63%.

### 4.2 Comparing the conceptions of and approaches to learning English

#### 4.2.1 Urban and rural students' conceptions of learning English

Descriptive statistics was analyzed to compare high school students' conceptions of learning English between students in the two investigated schools. As is shown in Tables 3 and 4, the mean value of Beijing students' conceptions of learning English was higher than that of Guizhou students in every dimension. Moreover, it is noticeable that Beijing and Guizhou students held conceptions of “Memorizing”, “Drills and Practice”, “Increasing Knowledge” and “Seeing in a New Way” to the similar degree. In addition, in terms of “Meeting the Requirements”, “Testing”, “Applying” and “Understanding”, the mean value of Beijing participants was much higher than that of Guizhou participants.

**Table 3:** Descriptive statistics of Beijing students' conceptions of learning English (*N* = 271).

**Factors**	**Min**	**Max**	**Mean**	**S.D**.	**Variance**
Meeting the requirements (MR)	1.00	5.00	3.40	1.11	1.23
Memorizing (Me)	1.00	5.00	3.09	0.97	0.94
Testing (Te)	1.00	5.00	2.94	1.10	1.20
Drills and practice (DP)	1.00	5.00	3.70	0.83	0.69
Increasing knowledge (IK)	1.00	5.00	3.80	0.85	0.72
Applying (Ap)	1.00	5.00	3.78	0.80	0.63
Understanding (Und)	1.00	5.00	3.78	0.85	0.72
Seeing in a new way (Se)	1.00	5.00	4.01	0.82	0.67

**Table 4:** Descriptive statistics of Guizhou students' conceptions of learning English (*N* = 345).

**Factors**	**Min**	**Max**	**Mean**	**S.D**.	**Variance**
Meeting the Requirements (MR)	1.00	5.00	2.73	0.88	0.77
Memorizing (Me)	1.00	5.00	2.95	0.71	0.51
Testing (Te)	1.00	5.00	2.60	0.78	0.61
Drills and Practice (DP)	2.00	5.00	3.64	0.54	0.29
Increasing Knowledge (IK)	2.00	5.00	3.65	0.63	0.39
Applying (Ap)	2.00	5.00	3.44	0.57	0.33
Understanding (Und)	2.00	5.00	3.54	0.61	0.37
Seeing in a New Way (Se)	2.00	5.00	3.84	0.58	0.33

#### 4.2.2 Urban and rural students' approaches to learning English

Descriptive statistical analysis was conducted to compare high school students' approaches to learning English between Beijing and Guizhou participants. The results are shown in Tables 5 and 6. Very similar to the results of conceptions, the mean value of Guizhou students' approaches to learning English was also lower than that of Beijing students in each dimension. And the standard deviation and variance of Guizhou students' approaches to learning English were much lower than those of Beijing students in each dimension. What's more, Beijing participants' mean values in terms of “Aim for Qualification”, “Fear of Failure”, “Intrinsic Interest and Commitment to Work” and “Minimizing Scope of Study” were significantly higher than those of Guizhou participants.

**Table 5:** Descriptive statistics of Beijing students' approaches to learning English (*N* = 271).

	**Min**	**Max**	**Mean**	**S.D**.	**Variance**
Aim for qualification	1.00	5.00	3.89	0.76	0.58
Fear of failure	1.00	5.00	3.63	0.88	0.77
Intrinsic interest and commitment to work	1.00	5.00	3.60	0.83	0.69
Minimizing scope of study	1.00	5.00	3.23	0.94	0.88
Memorization	1.00	5.00	2.96	1.04	1.09
Relating ideas and understanding	1.00	5.00	3.73	0.81	0.66

**Table 6:** Descriptive statistics of Guizhou students' approaches to learning English (*N* = 345).

	**Min**	**Max**	**Mean**	**S.D**.	**Variance**
Aim for qualification	2.00	5.00	3.59	0.55	0.30
Fear of Failure	1.50	5.00	3.35	0.66	0.44
Intrinsic interest and commitment to work	1.82	5.00	3.39	0.57	0.33
Minimizing scope of study	1.00	5.00	3.02	0.67	0.45
Memorization	1.00	5.00	2.79	0.78	0.61
Relating ideas and understanding	1.00	5.00	3.56	0.54	0.29

### 4.3 Comparing the correlations between the two constructs

Pearson's correlation analysis was conducted to reveal the relationships between the factors of these two surveys. With the results presented in Tables 7 and 8. Table 7 shows the correlation between urban students' conceptions of approaches to learning English. It can be drawn from the table that, for Beijing participants, their conceptions of “meeting the requirements” and “testing” were significantly related to surface motive (including “Aim for Qualification” and “Fear of Failure”) and surface strategies (including “Minimizing Scope of Study” and “Memorization”). “Meeting the requirements” was not significantly related to “intrinsic interest”; “testing” was not significantly associated with “intrinsic interest”, “relating ideas”, and “understanding”. In addition, “memorizing”, “drills and practice”, “increasing knowledge”, “applying”, “understanding” and “seeing in a new way” were all significantly correlated with each dimension of Beijing participants' conceptions of learning English.

**Table 7:** The correlations between Beijing students' responses to COLE and ATLE.

	**MR**	**Me**	**Te**	**DP**	**IK**	**AP**	**Und**	**Se**
AQ	0.28^***^	0.35^***^	0.23^***^	0.59^***^	0.63^***^	0.72^***^	0.71^***^	0.68^***^
FF	0.35^***^	0.47^***^	0.41^***^	0.57^***^	0.47^***^	0.42^***^	0.43^***^	0.37^***^
II-CW	0.07	0.247^***^	0.10	0.430^***^	0.55^***^	0.73^***^	0.67^***^	0.73^***^
MSS	0.41^***^	0.51^***^	0.63^***^	0.44^***^	0.38^***^	0.23^***^	0.27^***^	0.14^*^
Mem	0.29^***^	0.50^***^	0.43^***^	0.39^***^	0.38^***^	0.32^***^	0.37^***^	0.28^***^
RI-Und	0.13^*^	0.29^***^	0.10	0.53^***^	0.63^***^	0.70^***^	0.70^***^	0.72^***^

^*^p < 0.05, ^***^p < 0.001. MR, Meeting the Requirements; Me, Memorizing; Te, Testing; DP, Drills and Practice; IK, Increasing Knowledge; Ap, Applying; Und, Understanding; Se, Seeing in a New Way; AQ, Aim for Qualification; FF, Fear of Failure; II-CW, Intrinsic Interest and Commitment to Work; MSS, Minimizing Scope of Study; Mem, Memorization; RI-Und, Relating Ideas and Understanding.

**Table 8:** The correlations between Guizhou students' responses to COLE and ATLE.

	**MR**	**Me**	**Te**	**DP**	**IK**	**AP**	**Und**	**Se**
AQ	0.21^***^	0.32^***^	0.27^***^	0.41^***^	0.36^***^	0.48^***^	0.35^***^	0.39^***^
FF	0.25^***^	0.40^**^	0.33^***^	0.25^***^	0.22^***^	0.27^***^	0.23^***^	0.15^**^
II-CW	0.03	0.16^**^	−0.07	0.28^***^	0.35^***^	0.41^***^	0.44^***^	0.47^***^
MSS	0.45^***^	0.33^***^	0.58^***^	0.22^***^	0.15^**^	0.27^***^	0.10	−0.03
Mem	0.36^***^	0.45^***^	0.46^***^	0.30^***^	0.28^***^	0.37^***^	0.26^***^	0.11^*^
RI-Und	0.08	0.22^***^	−0.01	0.36^***^	0.42^***^	0.49^***^	0.54^***^	0.58^***^

^*^p < 0.05, ^**^p < 0.01, ^***^p < 0.001. MR, Meeting the Requirements; Me, Memorizing; Te, Testing; DP, Drills and Practice; IK, Increasing Knowledge; Ap, Applying; Und, Understanding; Se, Seeing in a New Way; AQ, Aim for Qualification; FF, Fear of Failure; II-CW, Intrinsic Interest and Commitment to Work; MSS, Minimizing Scope of Study; Mem, Memorization; RI-Und, Relating Ideas and Understanding.

Table 8 shows the correlation between Guizhou students' conceptions of and approaches to learning English. Very similar to the Beijing participants' results, Guizhou learners' conceptions of “meeting the requirements” and “testing”, were strongly associated to surface motives and surface strategies, generally speaking, surface approaches. What is different from Beijing participants is that approach of “minimizing scope of study”, was weakly related to conceptions of “understanding”, and negatively related to “seeing in a new way”, although the *p* value was not significant. It's also noticeable that Guizhou participants' conceptions of “testing” were significantly negatively related to approaches of “intrinsic interest”, which is another difference from Beijing participants.

Overall, the general finding was that students' conceptions of “testing” and “meeting the requirements” were significantly associated with surface approaches, including “surface motive” and “surface strategy”, corresponding to previous research findings. Moreover, conceptions of “memorizing”, “drills and practice”, “increasing knowledge”, “applying”, “understanding” and “seeing in a new way” were all strongly correlated with both surface approaches and deep approaches, among which “memorizing” ought to be surface conceptions according to previous research findings. What has been found is a little different from previous findings, and the reasons remain to be explored in the following discussion part.

### 4.4 *T*-test analysis

To investigate possible differences between the learners' conceptions of and approaches to learning English between urban and rural areas, independent sample *t*-tests were conducted. As Tables 9 and 10 indicate, there was a significant difference in terms of “Meeting the Requirements (MR)”, “Testing (Te)”, “Increasing Knowledge (IK)”, “Applying (Ap)”, “Understanding (Und)”, “Seeing in a New Way (Se)”, “Aim for Qualification (AQ)”, “Fear of Failure (FF)”, “Intrinsic Interest and Commitment to Work (II-CW)”, “Minimizing Scope of Study (MSS)”, “Memorization (Mem)”, and “Relating Ideas and Understanding (RI-Und)” between the urban and rural students. It is also noticeable that in terms of “Memorizing (Me)” and “Drills and Practice (DP)”, there was no significant difference between those of urban and rural students.

**Table 9:** Independent sample *t*-tests of Beijing and Guizhou students' COLE.

	**Levene's test for equality**		***T*** **-test for equality of means**						
	**F**	**Sig**.	***t***	**df**	**Sig. (two-tailed)**	**Mean difference**	**Std. error difference**	**95% confidence interval**	
								**Lower**	**Upper**
MR	16.71	0.000	8.21	504.99	0.000	0.68	0.08	0.51	0.84
Me	18.61	0.000	1.97	480.14	0.050	0.14	0.07	0.00	0.28
Te	25.55	0.000	4.33	468.83	0.000	0.34	0.08	0.19	0.50
DP	56.80	0.000	1.01	442.15	0.313	0.06	0.06	−0.06	0.17
IK	32.80	0.000	2.47	480.84	0.014	0.15	0.06	0.03	0.27
AP	26.12	0.000	5.93	472.15	0.000	0.34	0.06	0.23	0.45
Und	39.67	0.000	3.82	470.20	0.000	0.23	0.06	0.11	0.35
Se	18.81	0.000	2.90	465.35	0.004	0.17	0.06	0.05	0.29

MR, Meeting the Requirements; Me, Memorizing; Te, Testing; DP, Drills and Practice; IK, Increasing Knowledge; Ap, Applying; Und, Understanding; Se, Seeing in a New Way.

**Table 10:** Independent sample *t*-tests of Beijing and Guizhou students' ATLE.

	**Levene's test for equality**		***T*** **-test for equality of means**						
	**F**	**Sig**.	***t***	**df**	**Sig. (two-tailed)**	**Mean difference**	**Std. error difference**	**95% confidence interval**	
								**Lower**	**Upper**
AQ	28.73	0.000	5.47	472.44	0.000	0.30	0.06	0.19	0.41
FF	21.91	0.000	4.27	488.26	0.000	0.27	0.06	0.15	0.40
II-CW	32.19	0.000	3.46	457.52	0.001	0.20	0.06	0.09	0.32
MSS	22.95	0.000	3.16	472.19	0.002	0.21	0.07	0.08	0.35
Mem	9.96	0.002	2.197	485.86	0.028	0.17	0.08	0.02	0.32
RI-Und	41.59	0.000	3.01	444.20	0.003	0.17	0.06	0.06	0.29

AQ, Aim for Qualification; FF, Fear of Failure; II-CW, Intrinsic Interest and Commitment to Work; MSS, Minimizing Scope of Study; Mem, Memorization; RI-Und, Relating Ideas and Understanding.

### 4.5 Predictive relations analysis

A stepwise regression analysis was conducted to establish a regression model to describe the specific relationship between learners' conceptions of and approaches to learning English. The COLE factors were the predicators, while the ATLE factors were outcome variables. Tables 11 and 12 show the results of stepwise regression model for predicting Beijing and Guizhou participants' approaches to learning English. The general finding is that for Beijing participants, conceptions of “Applying”, “Testing”, and “Drills and Practice” played powerful roles in predicting learners' approaches to learning English. While for Guizhou participants, the conceptions of “Applying”, “Testing”, and “Understanding” were the strongest factors to predict students' approaches to learning. It is also interesting to find that factor “applying” made significant predictions for all the factors of approaches to learning English for both Beijing and Guizhou participants. It indicated that this factor played an overwhelmingly important role in predicting learners' approaches to learning English, including surface motives, surface strategies, deep motives, and deep strategies.

**Table 11:** Stepwise regression model for predicting Beijing students' ATLE.

**Variables**	**Factors**	**B**	**S.E**.	**β**	**T**	**Sig**.	**R**
AQ	AP	0.32	0.07	0.33	4.85^***^	0.000	0.80
	DP	0.25	0.04	0.28	6.32^***^	0.000	
	Und	0.18	0.06	0.20	3.05^**^	0.003	
	Se	0.14	0.06	0.15	2.31^*^	0.022	
	Constant	0.52	0.16		3.26^***^	0.001	
FF	DP	0.36	0.07	0.34	5.27^***^	0.000	0.63
	AP	0.30	0.06	0.27	4.97^***^	0.000	
	Te	0.17	0.05	0.21	3.58^***^	0.000	
	Constant	0.69	0.24		2.90^**^	0.004	
II-CW	AP	0.41	0.07	0.39	5.91^***^	0.000	0.77
	Se	0.37	0.07	0.36	5.43^***^	0.000	
	DP	0.11	0.04	0.11	2.55^*^	0.011	
	Constant	0.17	0.18		0.90	0.368	
MSS	Te	0.48	0.04	0.56	11.79^***^	0.000	0.66
	IK	0.25	0.05	0.22	4.70^***^	0.000	
	Constant	0.88	0.21		4.28^***^	0.000	
Mem	Te	0.33	0.05	0.33	6.76^***^	0.000	0.58
	AP	0.33	0.06	0.24	5.04^***^	0.000	
	Me	0.25	0.06	0.23	4.39^***^	0.000	
	Constant	0.09	0.23		0.39	0.698	
RI-Und	Se	0.32	0.07	0.33	4.79^***^	0.000	0.79
	DP	0.20	0.04	0.20	4.54^***^	0.000	
	AP	0.22	0.07	0.22	3.12^**^	0.002	
	Und	0.17	0.06	0.18	2.65^**^	0.009	
	Constant	0.21	0.18		1.17	0.24	

^*^p < 0.05, ^**^p < 0.01, ^***^p < 0.001. N = 271. MR, Meeting the Requirements; Me, Memorizing; Te, Testing; DP, Drills and Practice; IK, Increasing Knowledge; Ap, Applying; Und, Understanding; Se, Seeing in a New Way; AQ, Aim for Qualification; FF, Fear of Failure; II-CW, Intrinsic Interest and Commitment to Work; MSS, Minimizing Scope of Study; Mem, Memorization; RI-Und, Relating Ideas and Understanding.

**Table 12:** Stepwise regression model for predicting Guizhou students' ATLE.

**Variables**	**Factors**	**B**	**S.E**.	**β**	**T**	**Sig**.	**R**
AQ	AP	0.24	0.05	0.25	4.49^***^	0.000	0.58
	DP	0.17	0.05	0.17	3.21^***^	0.001	
	Te	0.17	0.03	0.24	4.95^***^	0.000	
	Se	0.21	0.05	0.22	3.84^***^	0.000	
	Constant	0.92	0.21		4.33^***^	0.000	
FF	Me	0.25	0.05	0.27	4.73^***^	0.000	0.47
	Te	0.19	0.05	0.22	4.11^***^	0.000	
	Und	0.17	0.06	0.16	3.14^**^	0.002	
	Constant	1.53	0.22		6.92^***^	0.000	
II-CW	Se	0.27	0.06	0.27	4.61^***^	0.000	0.53
	Und	0.20	0.06	0.21	3.51^***^	0.001	
	AP	0.15	0.06	0.15	2.56^*^	0.011	
	Constant	1.16	0.196		5.89^***^	0.000	
MSS	Te	0.40	0.05	0.47	8.95^***^	0.000	0.62
	AP	0.21	0.05	0.18	4.04^***^	0.000	
	MR	0.12	0.04	0.15	2.90^**^	0.004	
	Constant	0.95	0.19		5.03^***^	0.000	
Mem	Me	0.22	0.05	0.21	4.09^***^	0.000	0.60
	Te	0.30	0.05	0.32	6.54^***^	0.000	
	AP	0.25	0.07	0.20	3.66^***^	0.000	
	Und	0.16	0.06	0.14	2.51^*^	0.013	
	Constant	0.03	0.23		0.13	0.897	
RI-Und	Se	0.33	0.05	0.36	6.73^***^	0.000	0.65
	Und	0.22	0.05	0.25	4.71^***^	0.000	
	AP	0.15	0.05	0.16	3.02^**^	0.003	
	Constant	1.00	0.17		6.01^***^	0.000	

^*^p < 0.05, ^**^p < 0.01, ^***^p < 0.001. N = 345. MR, Meeting the Requirements; Me, Memorizing; Te, Testing; DP, Drills and Practice; IK, Increasing Knowledge; Ap, Applying; Und, Understanding; Se, Seeing in a New Way; AQ, Aim for Qualification; FF, Fear of Failure; II-CW, Intrinsic Interest and Commitment to Work; MSS, Minimizing Scope of Study; Mem, Memorization; RI-Und, Relating Ideas and Understanding.

Last but not least, the results also revealed that students' higher-level conceptions of learning English can significantly predict students' higher-level and lower-level learning approaches, while lower-level conceptions of learning English can only predict the lower-level learning approaches. For example, “Testing” can only predict the “Surface Motive” and “Surface Strategy” of senior high school English learners in Beijing and Guizhou. It shows that the higher-level conceptions of learning can more comprehensively affect learners' learning motivation and learning strategies in the process of learning English by urban and rural senior high school students. This is an important and innovative finding, as previous studies most proved that learners' surface approaches were often related with their lower-level conceptions of learning.

## 5 Discussion

### 5.1 Comparing urban and rural students' COLE and ATLE

The results of descriptive statistics analysis revealed that in term of “Meeting the Requirements (MR)”, “Testing (Te)”, “Applying (Ap)”, “Aim for Qualification (AQ)”, and “Fear of Failure (FF)”, Beijing participants' mean values were significantly higher than those of Guizhou participants. It might be caused by the educational gap between Beijing and Guizhou, where the students in Beijing study English earlier and enjoy more language resources than the students in Guizhou. And as for “Memorizing (Me)”, “Drills and Practice (DP)”, “Increasing Knowledge (IK)”, “Understanding (Und)”, “Seeing in a New Way (Se)”, “Intrinsic Interest and Commitment to Work (II-CW)”, “Minimizing Scope of Study (MSS)”, “Memorization (Me)”, and “Relating Ideas and Understanding (RI-Und)”, Beijing and Guizhou learners showed similar values without significant differences. It was also interesting to find that for both Beijing and Guizhou learners, their mean scores of higher-level conceptions (“Drills and Practice”, “Increasing Knowledge”, “Applying”, “Understanding”, and “Seeing in a New Way”) were all higher than the mean scores of their lower-level conceptions (“Meeting the Requirements”, “Memorizing”, and “Testing”) of learning English. It indicated that both Beijing and Guizhou students tended to hold higher-level conceptions of learning English rather than lower-level conceptions of learning English. The results of correlation verified the significant interrelationship between learners' conceptions of and approaches to English learning, basically corresponding to the previous findings (Chin and Brown, 2000; Chiou et al., 2013; Yang et al., 2019). Previous findings have generally found that lower-level conceptions of learning tended to positively predict surface approaches, while higher-level conceptions of learning were associated with deep approaches to learning (Chiou and Liang, 2012; Yang et al., 2019; Umapathy et al., 2020).

### 5.2 Comparing the predictive roles of learners' COLE for ATLE

#### 5.2.1 Predictive role of “Memorizing”

In this research, it was found that for both urban and rural students, the conception of “Memorizing”, as a lower-level type of conceptions to learning, was significantly associated with both surface and deep approaches, which is different from the findings that lower-level conceptions of learning only related with surface approaches to learning (Lee et al., 2008; Liang et al., 2010, 2015; Shen et al., 2016). Thus, the special role of “Memorizing” as a conception of learning influencing learners' approaches, remains to be further explored. Although viewing English learning as “Memorizing” could probably limit learners' use of certain strategies during language learning, learners with positive perceptions of “memorizing” can be more inclined to adopting a range of strategies (Zheng et al., 2016).

Memorizing plays a significant role in the process of language learning, especially for Chinese language learners (Gu and Johnson, 1996; Shichun, 2003). For example, most Chinese students do not believe in “naturally acquired words” and adhere to the view of “memorizing words” (Wenyu, 1998). Influenced by Confucian culture, Chinese students generally believe that memorization and repetition can promote understanding and improve learning (Cai, 2007). As the Chinese old saying goes, one can understand a book after he has read it hundreds of times. Previous research also found that in the process of learning English, the most commonly used learning strategies of Chinese students are “Memorizing” strategies (Jiongying, 2002). It has been proved that the language learning strategies of “memorizing” can significantly improve Chinese students' performance in English exams (Xia and Zhenghou, 2015). In a word, repetition and memorization serve as the foundation mastering a foreign language (Penner, 1995; Zheng et al., 2016).

#### 5.2.2 Predictive role of “Testing”

Based on the stepwise regression analysis, the second significant interesting finding concerns the positive association between “Testing” and learners' surface approaches to learning, including “Aim for Qualification (AQ)”, “Fear of Failure (FF)”, “Minimizing Scope of Study (MSS)”, and “Memorization (Mem)”. For both Beijing and Guizhou students, “testing” only predicted their surface approaches, corresponding to previous research findings (Chiou et al., 2013). English language learning and teaching in China has been oriented by examinations in Chinese education for a long time. Passing English tests was found to be most crucial motivation in English language learning, and it further influenced Chinese students' language learning strategies (Zheng et al., 2016).

The fundamental reason for the situation of exam-oriented English learning is the score-oriented evaluation system. High school students have to not only cope with the college entrance examination, but also deal with all kinds of weekly, monthly and various kinds of model tests. Teachers' salaries and achievements were also even evaluated by students' scores in the tests. Both teachers and students are suffering under this score-only evaluation system (Yang, 2016). Therefore, this may explain the reason why both rural and urban students' conceptions of English learning were test-oriented, and the conceptions of “testing” strongly predicted their surface approaches, including both surface motives and surface strategies. The exam-oriented education system has deeply shackled teachers' teaching behavior and students' learning motivation (Yang, 2016). The score-oriented evaluation system leads to the ignorance of the evaluation of students' comprehensive quality and ability, as well as their learning performance during the language learning process. The most effective measure to solve this problem is probably changing the evaluation system for assessing high school students' performance, from “formative evaluation” mode to “summative evaluation” mode.

#### 5.2.3 The predictive roles of other factors of COLE

This research also found that the conception of “Applying” predicted both surface approaches (including surface motives and surface strategies) and deep approaches (including deep motives and deep strategies) for both Beijing and Guizhou students, indicating the overwhelming role of “Applying” during students' English learning. Chomsky believed that human language learning is by no means a simple process of imitation and memory, but a process of creative use of language (Chomsky, 1968). Language learning must not stay at the level of simple imitation, memorization and recitation, but should focus on applying the language flexibly and creatively (You, 2001). However, the cultivation of flexible language applying ability was never easy to achieve, and it must be based on a solid language foundation and a long-term process of repeated drills and practice (You, 2001). As the Chinese old saying goes, “practice makes perfect”.

Another interesting finding was that Beijing students' “surface motive”, “deep motive” and “deep strategy” were strongly associated with the conceptions of “drills and practice”, while Guizhou students' learning approaches related with “drills and practice” was only in the “Surface Motive” dimension. The possible explanation was that urban students have more opportunities to practice English than rural students, as the urban students have more access to English-practicing resources. For example, according to the large-scale research conducted by Xu (2020), Chinese urban students have more opportunities to take foreign teachers' classes and learn extra-curricular teaching materials to practice, while most rural students do not participate in extracurricular English learning, and they mainly study the contents of textbooks. In addition, another possible explanation could be that urban students have more online opportunities to practice English due to the widening digital divide between urban and rural areas of China (Song et al., 2020).

According to the stepwise regression results, for Guizhou students, the conception of “Understanding” was found to predict both surface approaches and deep approaches, including “Fear of Failure”, “Intrinsic Interest and Commitment to Work”, “Memorization” and “Relating Ideas and Understanding”. The current situation of Chinese rural education is relatively backward compared with urban education, and English is the biggest “weakness” of rural students among all the school subjects. In rural areas, students rarely speak English, and their ability to use language is very poor, most of whom even don't understand the content in the textbook, let alone for them to master what the teacher has taught in the classes (Wang, 2014). Thus, for Chinese rural students, the most important thing in their English learning is “Understanding”, and this can perhaps explain why the conception of “Understanding” predicted most of the dimensions of approaches to learning English of Guizhou students, playing a dominate role besides “Applying” and “Testing”.

### 5.3 The role of social-economic-cultural background

Family socio-economic status has a significant impact on students' learning, and students from families with high socio-economic status are more likely to perform better in schoolwork (Orr, 2003). A family's socio-economic status was found to be a critical factor which strongly influenced their educational expectations on their children, and parents' expectations had strong effects on affecting their children's learning (Boonk et al., 2018). The higher parents' socio-economic status is, the greater their expectations are on their kids (Huang, 2017). According to previous large-scale quantitative research conducted in China, Chinese rural students' level was significantly lower than urban students in family socio-economic status (Zhao, 2011). And rural parents tend to hold weak expectations for their children's educational achievements, which significantly affects their children's learning performance (Tianhui, 2010). For instance, parents with higher socio-economic status would give more pressure on their children to perform better in learning English well, because they are more aware of the importance of learning English for their children's future social position (Zhao, 2011). Chinese urban parents often hold positive attitudes toward English learning, while rural parents are less aware of the significance of learning English and have relatively lower expectations on their children's English learning. Due to rural parents' low social status, limited power and few social relations, the expectations of rural parents to change their children's lives through education have been weakened, and their views toward educational have been even naturally internalized with the concept of “education is useless”, prompting them to give up their investment in their children's education (Zhao, 2011). Although some rural parents can realize the importance of English as a compulsory subject, they tend to reduce their expectations for their children's English learning due to the uncertainty of their children receiving a higher level of education and development in the future (Wang, 2014). This low expectation will imperceptibly affect children's English learning motivation and learning outcomes, namely “Rosenthal effect”, or called “Pygmalion effect” (Mau, 1995; Kaplan et al., 2001). Thus, all these could possibly explain why Beijing students' mean value score of the “meeting the requirements” and “surface motive” (including “aim for qualification” and “fear of failure”) was much higher than that of Guizhou students. This finding also corresponds to the previous findings that urban students' motivation level was significantly higher than that of rural students in China (Ma et al., 2021).

Cultural background has been found to significantly affect students' motivation, cognition and academic performance (Hu et al., 2018). Asian people, especially Chinese, believe that surface learning strategies can lead to understanding and mastery (Leung, 2001), while Westerners regard it as “rote learning” (Leung, 2001). Thus, it is possible that Chinese students may more often adopt surface strategies in pursuit of mastery goals than their Western counterparts do (Guo and Leung, 2021). However, even within the same country, different ethnic backgrounds may affect students' achievement goals and learning outcomes (Hau and Ho, 2008). As mentioned above, over 70% of the Guizhou participants were ethnic minorities and most of them were Miao students. It has been found in previous literature that Miao students' deep strategies failed to significantly predict their achievement, reminding teachers of the need to pay closer attention to Miao students' strategy use (Guo and Leung, 2021). What's more, minorities living in rural areas usually had to learn their own ethnic languages since they were young, which means English being a third language for them besides Mandarin and their native language. In the process of trilingual acquisition, there is a language loss. The interference of the first language and the second language affects the understanding of the third language, and most ethnic minority students will be disturbed by this negative language transfer (Li, 2012). And this reason may explain why Guizhou students' conception of “Understanding” plays an important role in predicting both surface approaches and deep approaches.

## 6 Conclusion

### 6.1 Major findings

The current study investigated urban and rural high school learners' conceptions of learning English and approaches to learning English. The results of exploratory factor analyses indicate that learners' conceptions of English language learning include eight factors while their approaches to learning English consist of four factors. Both the two instruments displayed similar factor structures as revealed by previous work (e.g., Tsai, 2004; Chiou and Liang, 2012; Zheng et al., 2016), and both showed satisfactory alpha reliability. Moreover, the results of correlation and regression analyses verified the significant interrelationship between learners' conceptions of and approaches to English learning, basically corresponding to the previous findings (Chin and Brown, 2000; Lee et al., 2008; Liang et al., 2010, 2015; Chiou et al., 2013; Shen et al., 2016; Yang et al., 2019; Umapathy et al., 2020).

Three interesting findings were identified in this research. First, the general finding is that for Beijing participants, conceptions of “Applying”, “Testing”, and “Drills and Practice” played powerful roles in predicting learners' approaches to learning English. While for Guizhou participants, the conceptions of “Applying”, “Testing”, and “Understanding” were the strongest factors to predict students' approaches to learning. Second, the factor “Applying (AP)” makes significant predictions for all factors of approaches to learning English, indicating that this factor played an overwhelmingly important role in Beijing and Guizhou learners' conceptions of learning, and could effectively predict most of the aspects of learners' approaches to learning English. Third, it is also noticeable that the conceptions of “testing” only predicted surface approaches (including surface motives and surface strategies) for both Beijing and Guizhou learners, indicating that the “testing” played an important role in Beijing and Guizhou learners' conceptions of learning, and could significantly predict learners' surface approaches to learning English. What's more, for both Beijing and Guizhou students, their deep approaches could only be positively predicted by higher-level conceptions of learning English, and their surface approaches could be predicted by both lower-level and higher-level conceptions of learning English.

### 6.2 Pedagogical implications

In the light of the potential contributions and practical implications for English language teaching, researchers could use the CLOE and ATLE surveys to explore students' conceptions of and approaches to learning English to gain deeper understandings for students learning. The first pedagogical implication is that teachers in high schools can pay more attention to cultivating students' higher-level conceptions (such as “Drills and Practice”, “Increasing Knowledge”, “Applying”, “Understanding” and “Seeing in a New Way”) which could more possibly lead to deep approaches (such as “intrinsic interest and commitment to work” and “relating ideas and understanding” in English learning to improve students' English learning outcomes and performances). The second implication is that the policy makers could consider weakening the examination-oriented evaluation criterion and attach more importance to quality education, paying attention to students' English learning process instead of the scores in exams. Finally, educational resources can be more reasonably allocated, providing more English learning resources and opportunities for rural students to practice and apply English, which may be realized first online and then to students in rural schools since it is challenged by human, material and financial resources. With the gap between urban and rural education being narrowed, the obvious significance can be achieved by promoting educational equity, which safeguards national quality and avoids “the intergenerational transmission of social disparities” (Huang, 2023), and which requires the joint efforts of our educators, researchers and policy makers.

### 6.3 Limitations and suggestions

The study also has some limitations. Firstly, the study only used quantitative questionnaire surveys to investigate senior high school students' conceptions of and approaches to learning English. Future studies in this area could use more qualitative research methods such as one-to-one interviews or focus groups to get a deeper understanding of students' conceptions of and approaches to leaning English in urban and rural areas of China.

Secondly, the study only investigated students at a senior high school level. To achieve a more comprehensive understanding of Chinese students' conceptions of and approaches to learning English in language education, more studies could be conducted among students at other education levels and in other cultural and social contexts. For instance, future research could also focus on students in primary school, middle school, and colleges. In addition, under the background of strengthening vocational education, future researchers could also compare the English learning between vocational school students and ordinary school students in China.

Thirdly, the study only investigated students in Beijing and Guizhou, with the former being the capital city of China, and the latter being one of the most underdeveloped areas of China. Future studies could broaden the research scope and pay attention to more areas in urban and rural areas of China, which are not so typical and extreme cases like Beijing and Guizhou. For example, in China, there are not only urban areas or rural areas, but also urban-rural fringe areas. Perhaps future research could also focus on the comparison among students in urban, rural and urban-rural fringe areas.

## Data availability statement

The original contributions presented in the study are included in the article/supplementary material, further inquiries can be directed to the corresponding author.

## Ethics statement

The studies involving humans were approved by Changshun High School, Guizhou High School Attached to Beijing Normal University, Haidian District, Beijing, China. The studies were conducted in accordance with the local legislation and institutional requirements. Written informed consent for participation in this study was provided by the participants' legal guardians/next of kin. Written informed consent was obtained from the individual(s), and minor(s)' legal guardian/next of kin, for the publication of any potentially identifiable images or data included in this article.

## Author contributions

HF: Formal analysis, Writing – review & editing, Writing – original draft. HL: Resources, Writing – original draft, Writing – review & editing.
